# Preparation of Carbon Nanotube-Sulfur Nanohybrid Materials with Elemental Sulfur as a Feedstock

**DOI:** 10.1021/acsomega.5c12163

**Published:** 2026-01-26

**Authors:** Shang-Yu Tsai, Ying-Ling Liu

**Affiliations:** Department of Chemical Engineering, 106090 National Tsing Hua University, No. 101, Sec. 2, Kuang-Fu Road, Hsinchu 300044, Taiwan

## Abstract

Based on the concept of green chemistry and synthesis of new functional materials, elemental sulfur is employed as the feedstock for the preparation of nanohybrid materials of carbon nanotubes (CNT) and sulfur. Referring to the radical reaction-based inverse vulcanization process, 2 reaction routes are conducted in this work. In the first approach elemental sulfur is directly reacted with CNTs through the addition reaction between sulfur radicals and the C=C bonds of CNT surfaces. The obtained CNT-sulfur nanohybrids possess 23.2 wt % polysulfide segments chemically bonded to the outer surfaces of CNTs. The second route involves 2-step reactions, including the incorporation of poly­(glycidyl methacrylate) (PGMA) to CNTs through a surface-initiated atom transfer radical polymerization followed by an inverse vulcanization reaction between the PGMA-functionalized CNTs and elemental sulfur. 31.3 wt % polysulfide segments are chemically bonded to the PGMA-functionalized CNTs. The CNT-sulfur nanohybrids are subjected to metal ion absorption. The sample from route 2 exhibits an absorption capacity of 16 mg g^−1^ for Fe^3+^, which is several times higher than the values reported for sulfur polymers from inverse vulcanization. The inverse vulcanization-like process provides an effective platform for the synthesis of CNT-sulfur nanohybrids exhibiting high absorption capacity for metal ions.

## Graphical abstract

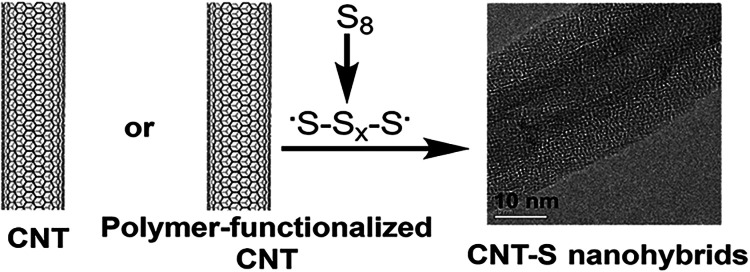

## Introduction

Nanohybrids are composed of two different kinds of materials mixed in nanoscale and usually exhibit attractive and integrated properties of the individual components.^1^ Carbon nanotubes (CNTs) are one of the attractive and widely studied carbon nanomaterials.^2^ Their hybrids with polymers,^3^ ceramics,^4^ and metals^5^ have received massive investigation on both material preparation and applications. Moreover, integration of CNTs with sulfur in one material received much attention due to their application potentials in lithium−sulfur batteries and others.^6–11^ Physical hybridization between CNTs and sulfur was conducted in prior studies. Taking the advantages of open-ended CNTs, Fujimori et al.^6^ prepared CNT-S nanohybrids through diffusing linear sulfur chains into CNTs. A similar technique was also conducted by Sysoev et al. in preparation of sulfur-filled CNTs, which was applied to real-time detection of NO_2_ in air.^11^ Sun et al.^7^ simply blended elemental sulfur, CNTs, and graphene through a solvent-mediated method. The carbon nanomaterials served as a sulfur host through the formation of a 3-dimensional framework. The method was further applied to porous CNTs for the fabrication of sulfur-absorbed CNT nanohybrids.^8^ Lin et al.^9^ used oxidized CNTs possessing various open-ring sizes to encapsulate sulfur in the resulting CNT-S nanohybrids. On the other hand, chemically bonding sulfur chains onto the CNTs surface could be interesting. Baratta et al.^10^ successfully thiolated the CNTs surface with –SH groups using elemental sulfur as a reagent through a UV-activated process. The presence of C–SH groups was verified with XPS and Raman spectral analysis. Nevertheless, the sulfur contents of the products were quite limited compared with the CNT-S nanohybrids prepared with physical methods, as only –SH groups rather than polysulfide segments were bonded to the CNTs surfaces. Hence, the product could be considered as a surface-thiolated CNT rather than a CNT-S nanohybrid.^10^ In 2025, Deng et al.^12^ carried out inverse vulcanization (IV) of sulfur and 1,3-diisopropylbenzene (DIB) in the pores of hollow porous carbons. Sulfur polymers were formed in the confined pores of the carbon materials. The prepared CNT-S nanohybrids were applied to lithium batteries,^8,9^ gold recovery,^10^ and NO_2_ detection.^11^

In this work, convenient chemical reaction routes for the preparation of CNT-S nanohybrids, in which polysulfide segments are chemically bonded to CNTs, are reported. CNTs possessing unsaturated C=C bonds at surfaces exhibit chemical reactivity toward radicals.^3,13–15^ The radical-mediated reactions have been utilized in the surface modification of CNTs with organic moieties and polymer chains. On the other hand, the IV process,^16^ which involves the radical-based reactions between an unsaturated organic compound and elemental sulfur, has been widely studied.^17^ The abovementioned reactions are integrated to develop chemical routes for the synthesis of CNT-S nanohybrids in this work. The first attempt is conducted through the direct reaction between CNTs and elemental sulfur by means of an IV-similar process. The other reaction route involves 2-step reactions: surface functionalization of CNTs with epoxide-containing polymer chains and the reaction between the functionalized CNTs and elemental sulfur through IV processes in which the epoxide groups^18^ are utilized as the organic moieties in reacting with sulfur radicals. In addition to the developments of chemical routes for the preparation of CNT-S nanohybrids, the application potential of the obtained materials in the removal of metal ions is also examined, as utilization of the S-containing polymers from IV processes in metal ion removal has been widely reported.^19–21^

## Experimental Section

### Materials

Sulfur (S_8_, sublimed powder, 99.5%), 1-bromoethylbenzene (BEB), and iron­(III) chloride (FeCl_3_) were purchased from Alfa Aesar. Multiwalled carbon nanotubes with diameters of 10−50 nm and lengths of 1−25 μm were received from Carbon Nanotube Co., Ltd. (Korea). The received samples were washed with dimethylformamide (DMF) prior to use. 2,2′-Bipyridyl, copper bromide­(I), glycidyl methacrylate (GMA), and *N*,*N*,*N*′,*N*″,*N*′″-pentamethyldiethylenetriamine (PMDTA) were purchased from Sigma-Aldrich. HPLC-grade DMF and tetrahydrofuran (THF) were purchased from TEDIA and used as received.

### Instrumental Methods

A Renishaw InVia Raman spectrometer was utilized in recording the Raman spectra with a He−Ne laser at 632.8 nm as the radiation source. The peak intensity was calibrated with baseline correction for the calculation of the peak intensity ratios. X-ray photoelectron spectroscopy (XPS) of the PHI 5000 Versa Probe II instrument (from ULVAC-PHI, Inc.) equipped with a Mg−Kα line as the radiation source was employed in XPS analysis. Differential scanning calorimetry (DSC) thermograms were recorded with a TA-Q20 instrument (Thermal Analysis Co.) in a temperature range from 50 to 140 °C and at a heating rate of 10 °C min^−1^. Thermogravimetric analysis (TGA) was performed using a TA-Q50 instrument under a nitrogen atmosphere from 50 to 800 °C at a heating rate of 10 °C min^−1^. High-resolution transmission electron microscopy (HRTEM) micrographs were obtained with a JEOL JEM-2010 HRTEM instrument equipped with a liquid nitrogen cooling stage. CNT samples were dispersed in THF. After being stirred at room temperature for 24 h, the solution was dropped on a 200 mesh TEM grid and then dried under vacuum at room temperature for 48 h. Quantitative measurements of the Fe^3+^ concentrations of the aqueous samples were conducted with an optical emission spectroscopy equipped with an inductively coupled plasma as the atomizer (Thermo Scientific, iCAP 7200 Duo ICP-OES). Calibration curve method with external standards was employed.

### Synthesis of Carbon Nanotube-Sulfur Nanohybrid CNT-S-R1

CNT (150 mg) and elemental sulfur (3000 mg) were charged into a glass reactor (50 mL). The mixture was reacted at 165 °C for 18 h. The resulting product was washed with THF several times and then dried at 60 °C under vacuum to give the reaction product of CNT-S-R1.

### Synthesis of Carbon Nanotube-Sulfur Nanohybrid CNT-S-R2

CNT (210 mg) and BEB (490 mg) were dispersed in THF (30 mL). The solution was charged into a glass reactor. After 2,2′-bipyridyl (43.4 mg) and copper bromide (I) (21 mg) were added, the reaction system was purged with argon for 30 min and then applied to the freeze-thaw degassing process. After being reacted at 60 °C for 24 h, the CNTs were collected with a centrifuge, washed with THF, and dried at 60 °C under vacuum to give the reaction product of CNT-BEB.

CNT-BEB (100 mg) and GMA (1.6 g) were dispersed in DMF (10 mL). Copper bromide (I) (13.5 mg) and PMDTA (40 μL) were added to the solution. After being purged with argon and degassed with the freeze-thaw process, the solution was reacted at 60 °C for 24 h. The CNTs were collected with a centrifuge, washed with THF, and dried at 60 °C under vacuum to give the reaction product of CNT-PGMA.

CNT-PGMA (100 mg) and elemental sulfur (1000 mg) were charged into a glass reactor (50 mL). After being reacted at 165 °C for 18 h, the resulting product was washed with THF several times and dried at 60 °C under vacuum to give the reaction product of CNT-S-R2.

## Results and Discussion

The chemical routes for the synthesis of chemically bonded CNT-S nanohybrid materials are shown in Figure 1. In route 1, CNTs and elemental sulfur were mixed and reacted without the addition of any catalysts or solvents at 165 °C (S_8_ in its molten state) for 18 h. CNTs without oxidative pretreatments were used as the raw materials since the radical addition reaction to the unsaturated C=C bond of CNTs was proposed. Oxidative pretreatments might be negative for the proposed addition reaction due to the reduction of the density of C=C bonds. In route 2, poly­(glycidyl methacrylate) (PGMA) chains were first chemically bonded to CNT surfaces through surface-initiated atom transfer radical (SI-ATRP) polymerization by means of the method reported in the prior publication.^22^ The obtained product of PGMA-modified CNT (CNT-PGMA) was then reacted with molten elemental sulfur. As the CNT outer surfaces were mostly covered with PGMA chains, the reaction between CNT-PGMA could mostly be conducted through the PGMA chains, rather than the unsaturated C=C bonds of CNTs, with sulfur radicals since epoxide groups have been demonstrated as effective reagents for IV processes.^18^ The CNT-S nanohybrid materials obtained through routes 1 and 2 are coded as CNT-S-R1 and CNT-S-R2, respectively.

**Figure 1:**
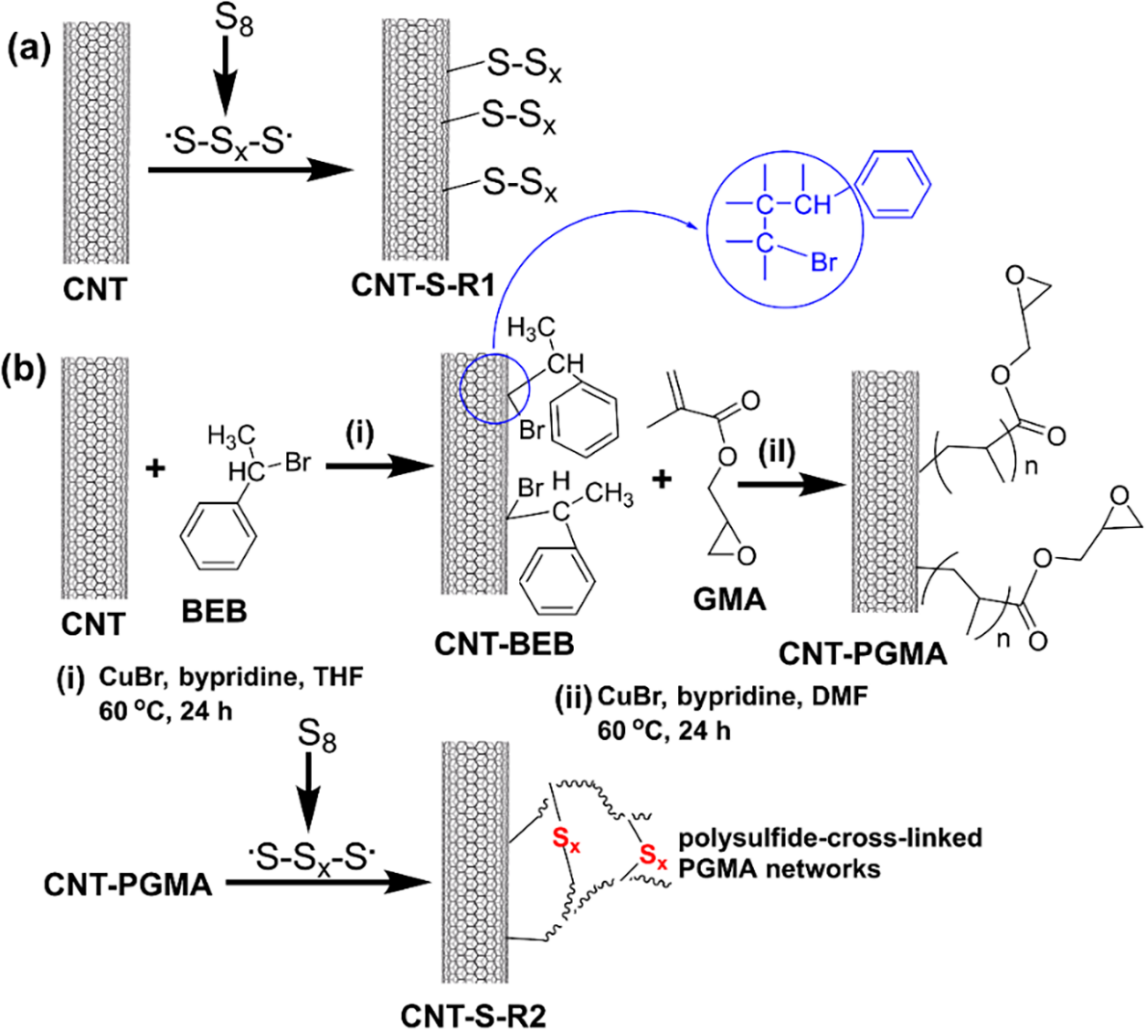
Reaction routes for the preparation of CNT-sulfur nanohybrid materials: (a) CNT-S-R1 and (b) CNT-S-R2.

Characterization of CNT-S-R1 is presented in Figure 2. In the Raman spectrum, the pristine CNTs exhibited 2 major signals at 1333 cm^−1^ (D band) and 1588 cm^−1^ (G band) with an intensity ratio (*I*_D_/*I*_G_) of about 0.97. After being reacted with elemental sulfur, CNT-S-R1 still exhibited 2 signals, nevertheless, with an increased *I*_D_/*I*_G_ value of 1.08, which could be attributed to the increased disorder (sp^3^) mode of carbon atoms associated with the sulfur radical addition to C=C bonds. The change in the *I*_D_/*I*_G_ is significant to support the chemical surface modification of CNTs, as a recent study on surface carboxylation of CNTs giving a change in *I*_D_/*I*_G_ ratio from 0.96 to 1.01.^23^ In addition to the D band and G band signals, CNT-S-R1 also exhibited signals at 148, 225, and 473 cm^−1^ being assigned to asymmetric S−S bending, symmetric S−S bending, and S−S stretching, respectively. Three signals were also found with elemental sulfur but not with CNTs, indicating CNT-S-R1 possessing sulfur segments. Moreover, compared with elemental sulfur, CNT- S-R1 exhibited an additional C−S signal at 518 cm^−1^ to indicate the occurrence of a chemical addition reaction between CNTs and sulfur radicals. The presence of sulfur in CNT-S-R1 has been verified with X-ray photoelectron spectroscopy (XPS). Sulfur signals were observed with CNT-S-R1, but not with CNTs, in both the wide-scan and the S_2p_ core-level XS spectra. Nevertheless, the signal of C−S bonds in sulfur−carbon nanotube materials was at about 163.6 eV,^24^ which could not be distinguished from the polysulfide signals in the S_2p_ core-level spectrum of CNT-S-R1. In C_1s_ core-level spectra, the pristine CNT exhibited a C−O signal in low intensity due to the presence of oxidative groups. This signal was also observed with CNT-S-R1 with an increased intensity, being attributed to the formation of C−S linkages. In differential scanning calorimetric (DSC) measurements, elemental sulfur showed 2 endothermic peaks at 113 and 119.5 °C being assigned as the melting behaviors of S_8_ orthorhombic crystals and monoclinic crystals, respectively. These 2 peaks were not observed with the DSC thermogram of CNT-S-R1, indicating this sample did not possess crystalline sulfur. Hence, the sulfur signals detected in the Raman and XPS spectra of CNT-S-R1 were from the chemically bonded sulfur segments rather than the physically absorbed S_8_ crystals. The sulfur weight fraction of CNT-S-R1 was determined with a thermogravimetric analysis to give the value of about 23.2 wt %. As the sulfur contents of sulfur polymers prepared from IV processes are above 50 wt %, the relatively small sulfur amount of CNT-S-R1 indicates that, in the reaction, CNTs served as a reagent rather than a cross-linking agent for elemental sulfur. The obtained product is sulfur-surface-functionalized CNTs rather than CNT-cross-linked sulfur networks.

**Figure 2:**
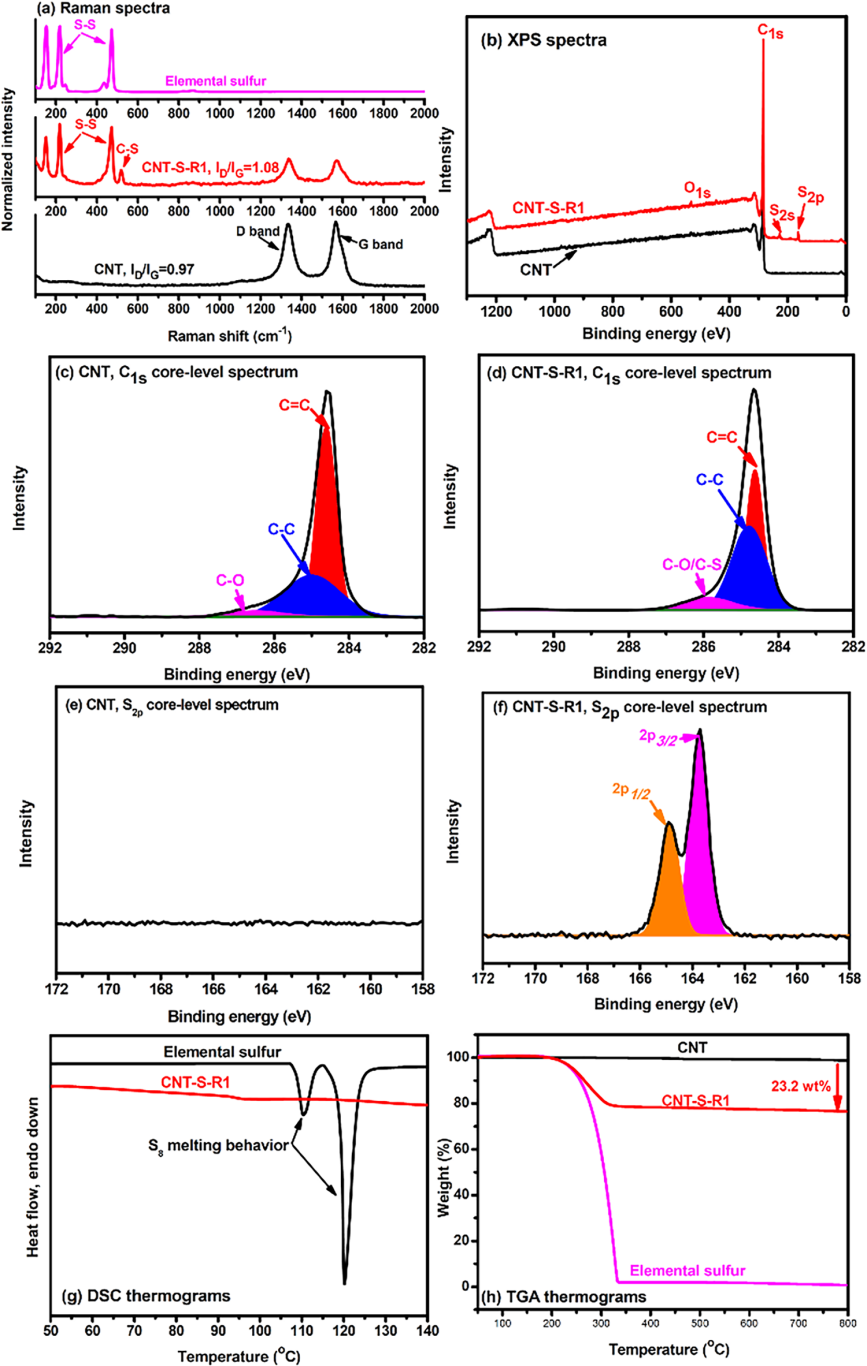
Spectral and thermal analysis on CNT and CNT-S-R1: (a) Raman spectra, (b) XPS wide-scan spectra, (c) C_1s_ core-level spectrum of CNT, (d) C_1s_ core-level spectrum of CNT-S-R1, (e) S_2p_ core-level spectrum of CNT, (f) S_2p_ core-level spectrum of CNT-S-R1, (g) DSC thermograms, and (h) TGA thermograms measured in air.

CNT-S-R2 was characterized in a similar manner (Figure 3). Incorporation of PGMA chains to CNT outer surfaces was conducted with 2-step reactions shown in Figure 1. In Raman spectroscopic analysis, CNT-PGMA showed an *I*_D_/*I*_G_ value of 1.11, which was higher than the value of 0.97 found with the pristine CNTs. Sulfur was incorporated to CNT-PGMA through an IV process, in which elemental sulfur reacted with glycidyl groups through the hydrogen abstraction reaction and thiol/epoxide addition reaction.^18^ The incorporated sulfur segments exhibited similar signals in the Raman spectrum of CNT-S-R2. It is noteworthy that CTN-S-R2 exhibited a relatively strong signal of C−S bonds at 518 cm^−1^, supporting that elemental sulfur performed the IV reactions rather than radical addition reaction to the C = C bonds of CNTs. The presence of sulfur in CNT-S-R2 was still observed with the S signals in the XPS wide-scan measurement. In the S_2p_ core-level spectrum, an additional signal at about 161.7 eV was observed and attributed to the bound thiol groups^26^ formed with the IV process between sulfur and PGA chains.^18^ Nevertheless, due to the tiny PGMA segments grafted on CNT-PGMA, characterization of the reaction between epoxide and elemental sulfur with FTIR measurements did not give strong and reliable evidence due to the nearly invisible absorption of epoxide groups. DSC thermogram of CNT-S-R2 did not show any endothermic peaks, supporting the crystalline sulfur-free feature of the prepared sample. CNT-PGMA showed a weight loss of 49.0 wt % (organic moieties) at 800 °C. The weight loss found with CNT-S-R2 was 65.0 wt % (PGMA + S). Hence, the compositions calculated for CNT-S-R2 were CNT: 35.0 wt %, PGMA: 33.7 wt %, and S: 31.3 wt %. CNT-S-R2 possessed a relatively higher sulfur content than did CNT-S-R1, as PGMA chains provided more reactive sites for sulfur radicals. The PGMA/sulfur polymers at the outer surface of CNT-S-R2 had a sulfur content of about 48.1 wt %, providing additional support to the performance of IV reactions between PGMA and elemental sulfur. Nevertheless, the PGMA chains in a confined environment (chemically tethered at CNT surfaces) might hinder the reaction between PGMA chains and elemental sulfur, since the sulfur content (48.1 wt %) was not as high as reported for the S-containing polymers (above 50 wt %) in IV processes. The sulfur contents of the CNT-S-R1 and CNT-S-R2 could be reasonable since the reactions only performed at the outer surfaces of CNTs. The amounts of sulfur being bonded to CNT surfaces are comparable to the fractions of modifying agents reported to CNT nanohybrids.^27–29^

**Figure 3:**
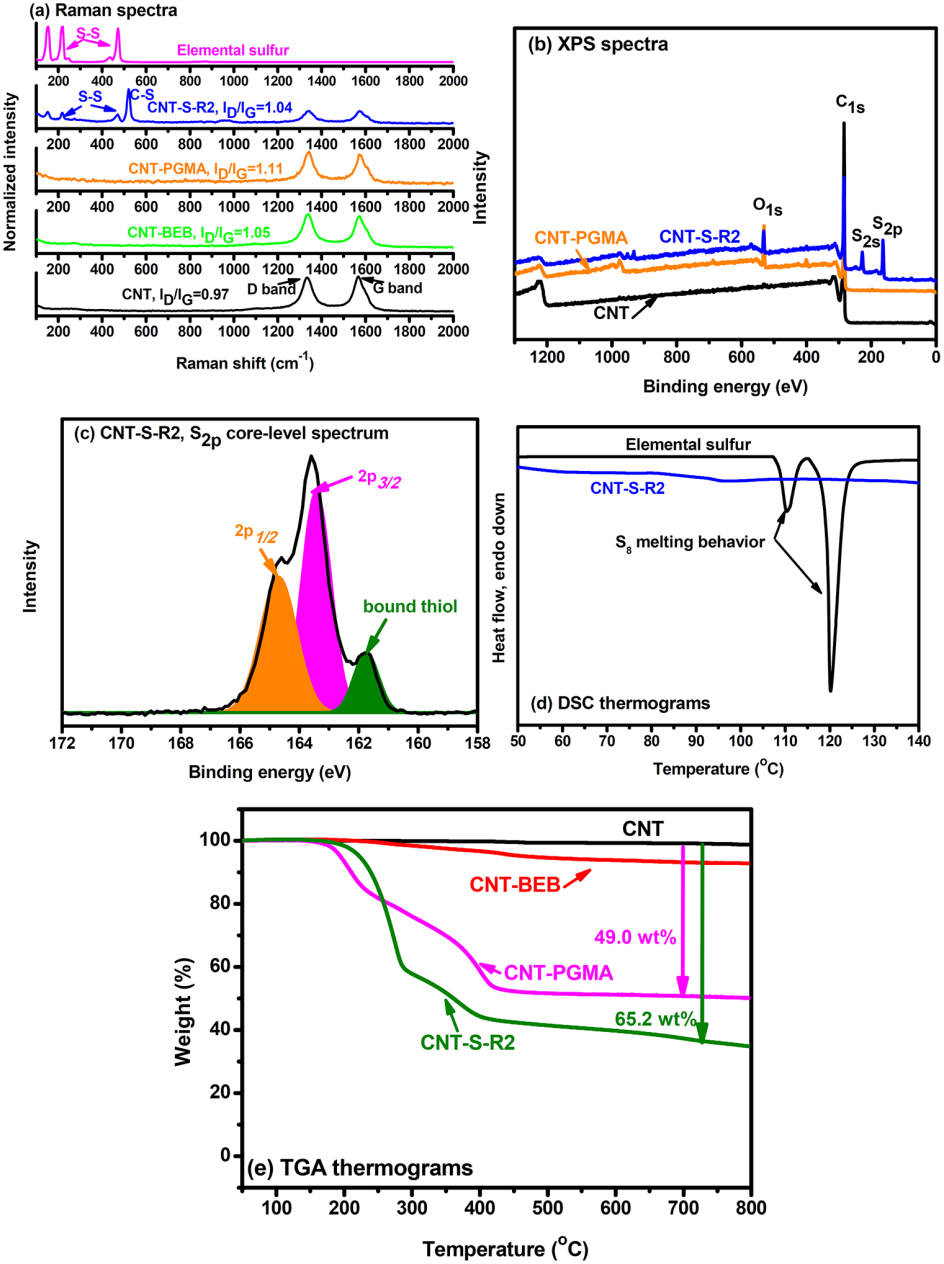
Spectral and thermal analysis on CNT-S-R2: (a) Raman spectra, (b) XPS spectra, (c) CNT-S-R2, S_2p_ core-level spectrum, (d) DSC thermograms, and (e) TGA thermograms measured inair.

Figure 4 collects the micrographs of the CNT samples recorded with high-resolution transmission electron microscopy (HRTEM). Continuous and smooth amorphous layers were observed at the outer surfaces of the functionalized CNTs, indicating the functionalization reaction was conducted uniformly. CNT-S-R1 showed an amorphous layer with a thickness of about 7 nm at the outer surface of CNT bundles, supporting the presence of a bonded sulfur layer. Nevertheless, the thickness of the measured sulfur layer might be relatively large and could not reasonably be compared with the sulfur content. Further examination revealed that the sulfur layers would expand with the irradiation time in the TEM measurements. The sulfur layer thickness probed at the beginning of irradiation was about 3.5 nm. During the HRTEM measurements, the interaction between sulfur segments and electrons resulted in rearrangements of the sulfur segments so as to contribute to the expansion of the sulfur layers. Similar results were also observed with CNT-S-R2, which exhibited an outer layer with a thickness of about 10 nm, which was much larger than the thickness of about 3 nm found with CNT-PGMA.

**Figure 4:**
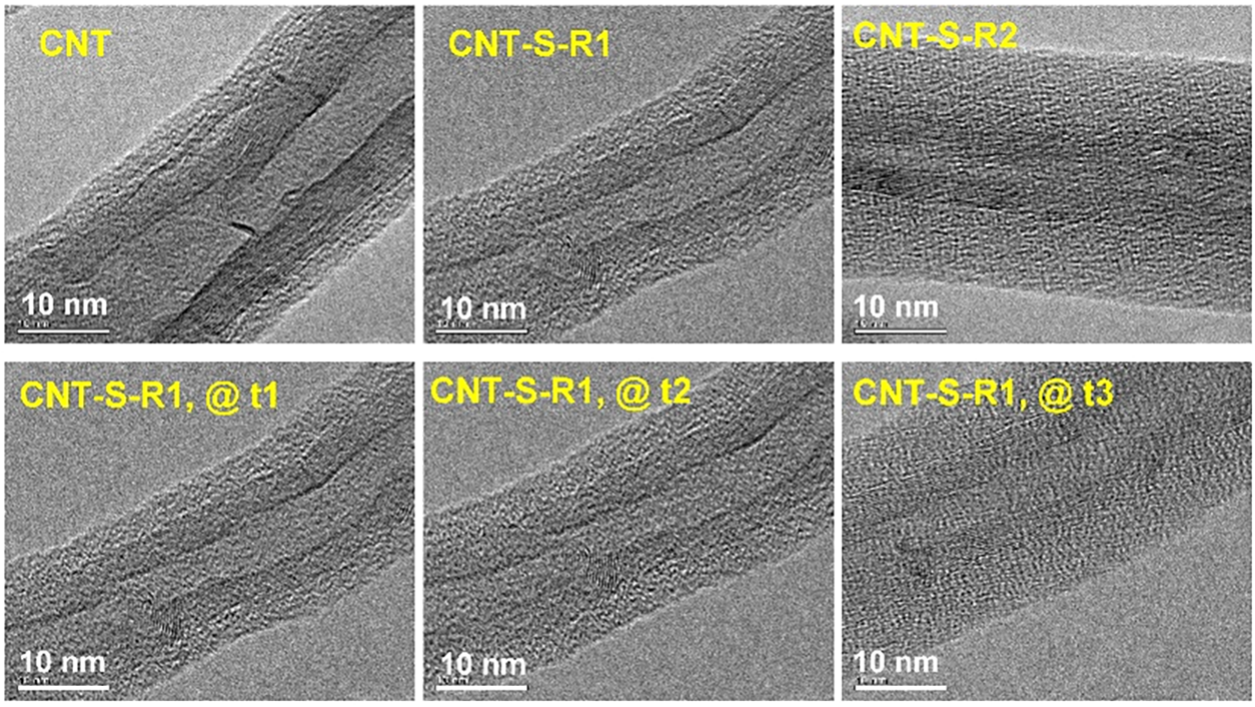
HRTEM micrographs recorded on the pristine CNTs and CNT-S nanohybrids (upper row). The micrographs collected in the lower row showing the pictures of CNT-S-R1 recorded at different irradiation times in the measurements, in which *t*1 < *t*2 < *t*3.

The presence of sulfur segments of the prepared CNT-S hybrid materials was further examined with the metal ion absorption, as sulfur-based materials have been widely applied to the absorption and removal of metal ions.^19–21^ In the tests, CNT samples (30 mg) were added to FeCl_3_ aqueous solutions (Fe^3+^ concentration: 97.6 ppm, 5.0 mL) and stirred at room temperature for 20 h. As shown in Figure 5, the Fe^3+^ concentrations in the after-test solutions were 59.2, 58.8, and 32.5 ppm for CNT, CNT-S-R1, and CNT-S-R2, respectively, supporting the absorption of Fe^3+^ ions. The neat CNTs still showed obvious Fe^3+^ absorption and removal from the aqueous solution. The result was similar to what was reported in literature,^30^ telling both physical absorption and chemical interaction through the oxygen-containing groups of the oxidized CNTs with metal ions contributing to the absorption. Nevertheless, as the surface of CNT-S-R1 is covered with polysulfide segments, the absorption of CNT-S-R1 with Fe^3+^ should be conducted with a sulfur−metal interaction rather than a CNT−metal interaction. With regret, both CNT and CNT-S-R1 showed similar Fe^3+^removal ability, and the contribution of the grafted polysulfide segment of CNT-S-R1 could not be clearly demonstrated. On the other hand, the relatively low Fe^3+^ concentration (32.5 ppm) in the after-testing solution with CNT-S-R2 is noteworthy. With surface modification, the Fe^3+^removal efficiency of CNT-S-R2́, compared with neat CNTs, was enhanced to 170%. The Fe^3+^ absorption capacity of CNT-S-R2 was calculated to be 16 mg g^−1^ in the test with such a low concentration of Fe^3+^ aqueous solution. The efficiency is much higher than the values of 0.8 mg g^−1^ and 0.28 mg g^−1^ reported to biomass-based sulfur polymers made with biomass-based agents.^31,32^ Incorporation of inverse vulcanization polymers on CNT surfaces provides an effective approach to the preparation of CNT-sulfur nanohybrid materials for high-efficiency metal ion removal. The preliminary examination on metal ion absorption was conducted with Fe^3+^. As sulfur-rich polymers have been applied to the absorption of other metal ions,^33^ the prepared CNT-sulfur nanohybrid might still possess the ability of absorption of other metal ions.

**Figure 5:**
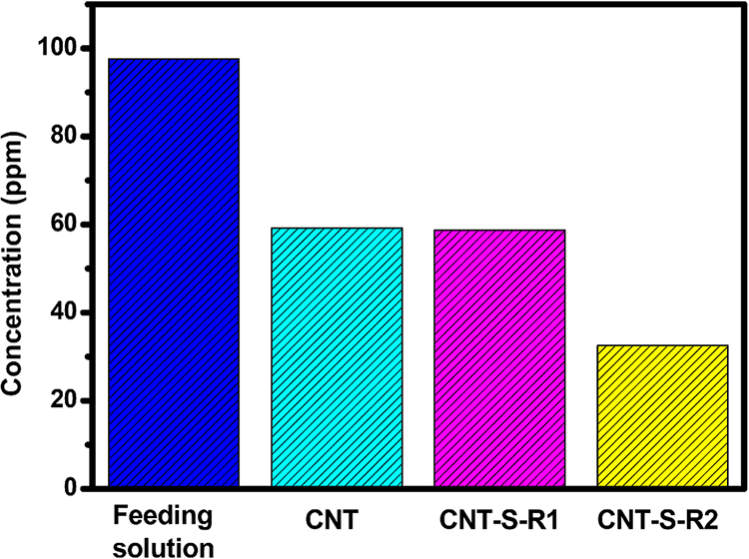
Fe^3+^ concentrations measured on the solutions in the metal ion absorption tests with the prepared CNT-S samples.

## Conclusions

Based on the inverse vulcanization-like reactions, elemental sulfur has been demonstrated as a suitable reactant for the synthesis of carbon nanotube-sulfur nanohybrid materials. The reactions could be performed on both the neat CNTs and the CNTs functionalized with IV reagents (in this work, PGMA chains). In addition, the high surface areas of the CNT-sulfur nanohybrids contributed to the high absorption capacities for metal ions compared with the values reported to other IV polymers. This work has extended the scope of utilization of elemental sulfur in material synthesis and explored an effective platform for the preparation of CNT-sulfur nanohybrids. Taking advantage of the high surface areas of the CNT-sulfur nanohybrids, high absorption capacities for metal ions have been recorded on the nanohybrid materials to warrant their potential applications. Moreover, as CNT-sulfur hybrid materials utilized in lithium−sulfur batteries are reported,^34,35^ the reported synthetic methods and the obtained materials might be potentially applied to this class of applications in future studies.
